# Recent amplification of microsatellite-associated miniature inverted-repeat transposable elements in the pineapple genome

**DOI:** 10.1186/s12870-021-03194-0

**Published:** 2021-09-18

**Authors:** Lianyu Lin, Anupma Sharma, Qingyi Yu

**Affiliations:** 1 Texas A&M AgriLife Research Center at Dallas, Texas A&M University System, Dallas, TX 75252 USA; 2 College of Life Science, Fujian Agriculture and Forestry University, Fuzhou, 350002 Fujian China

**Keywords:** Pineapple, Miniature inverted-repeat transposable elements, (TA) n dinucleotide microsatellite, *Ananas*

## Abstract

**Background:**

Miniature inverted-repeat transposable elements (MITEs) are non-autonomous DNA transposable elements that play important roles in genome organization and evolution. Genome-wide identification and characterization of MITEs provide essential information for understanding genome structure and evolution.

**Results:**

We performed genome-wide identification and characterization of MITEs in the pineapple genome. The top two MITE families, accounting for 29.39% of the total MITEs and 3.86% of the pineapple genome, have insertion preference in (TA) n dinucleotide microsatellite regions. We therefore named these MITEs *A. comosus* microsatellite-associated MITEs (Ac-mMITEs). The two Ac-mMITE families, Ac-mMITE-1 and Ac-mMITE-2, shared sequence similarity in the terminal inverted repeat (TIR) regions, suggesting that these two Ac-mMITE families might be derived from a common or closely related autonomous elements. The Ac-mMITEs are frequently clustered via adjacent insertions. Among the 21,994 full-length Ac-mMITEs, 46.1% of them were present in clusters. By analyzing the Ac-mMITEs without (TA) n microsatellite flanking sequences, we found that Ac-mMITEs were likely derived from *Mutator*-like DNA transposon. Ac-MITEs showed highly polymorphic insertion sites between cultivated pineapples and their wild relatives. To better understand the evolutionary history of Ac-mMITEs, we filtered and performed comparative analysis on the two distinct groups of Ac-mMITEs, microsatellite-targeting MITEs (mt-MITEs) that are flanked by dinucleotide microsatellites on both sides and mutator-like MITEs (ml-MITEs) that contain 9/10 bp TSDs. Epigenetic analysis revealed a lower level of host-induced silencing on the mt-MITEs in comparison to the ml-MITEs, which partially explained the significantly higher abundance of mt-MITEs in pineapple genome. The mt-MITEs and ml-MITEs exhibited differential insertion preference to gene-related regions and RNA-seq analysis revealed their differential influences on expression regulation of nearby genes.

**Conclusions:**

Ac-mMITEs are the most abundant MITEs in the pineapple genome and they were likely derived from *Mutator*-like DNA transposon. Preferential insertion in (TA) n microsatellite regions of Ac-mMITEs occurred recently and is likely the result of damage-limiting strategy adapted by Ac-mMITEs during co-evolution with their host. Insertion in (TA) n microsatellite regions might also have promoted the amplification of mt-MITEs. In addition, mt-MITEs showed no or negligible impact on nearby gene expression, which may help them escape genome control and lead to their amplification.

**Supplementary Information:**

The online version contains supplementary material available at 10.1186/s12870-021-03194-0.

## Background

Miniature inverted-repeat transposable elements (MITEs) are non-autonomous DNA transposable elements (TEs), transposing by a “cut and paste” mechanism. MITEs were first described in plant genomes [1] and later found in a wide range of organisms, including invertebrates [2, 3], vertebrates [4], fungi [5], and viruses [6]. MITEs are characterized by a small size (< 500 bp), a high copy number, a stable secondary structure, and terminal inverted repeats (TIRs) flanked by target site duplications (TSDs). MITEs exhibit the structural features of class II transposons and are considered as truncated derivatives of autonomous class II transposons [7, 8]. Unlike autonomous DNA transposons, MITEs lack coding capacity and transpose through transposases provided in *trans* by their related autonomous elements [9].

Independent studies showed that MITEs could be mobilized by transposases from their related elements [10, 11]. Homology restricted to the TIRs and the sub-terminal sequences between MITEs and their related elements could be sufficient for cross-mobilization [12]. However, MITEs are present at a much higher copy number than autonomous DNA transposons, which mobilize them and from which they are derived, suggesting that MITEs are particularly successful in avoiding genome control. Yang et al. revealed that the MITE lacks a motif repressing transposition in the autonomous element and contains internal sequences that enhance transposition [13]. The amplification of autonomous DNA elements may be limited by a self-regulatory mechanism, while MITEs could achieve high transposition activity by scavenging transposases encoded by distantly related and self-restrained autonomous DNA elements [13]. The small size of MITEs may also help them to avoid silencing by host genomes [14]. Although MITEs are abundant in eukaryotic genomes, only very few MITEs have been found to be active in transposition likely because they are subject to purifying selection [14].

MITEs are grouped into different MITE families based on their size, structure, and sequence similarity between their TIRs or TSDs and these of autonomous partners. The structural homogeneity of MITE families suggests that they arose from amplification of a few progenitor copies. Major MITE superfamilies, such as *Tc1*/*Mariner*, *PIF*/*Harbinger*, *h*AT, *Mutator*, and *CACTA*, have been described in plant genomes [9, 12, 13, 15]. Identification and classification of MITEs are mainly performed through searching sequences with TIR and TSD features. Bioinformatics programs, such as MITE-Hunter [16], MITE Digger [17], MITE tracker [18], detectMITE [19], and MAK [20], have been developed to identify MITEs from genome sequence databases.

MITEs are abundant in eukaryotic genomes and are thought to have a significant influence on the evolution of the host’s genome structure. MITEs can mediate genomic rearrangements through insertion, excision, chromosome breakage, and ectopic recombination [21]. In addition, MITEs can affect gene function and regulation by gene transduction, duplication, exon shuffling, and insertion in gene regulatory regions [21, 22]. MITEs can change host gene expression by generating small RNAs, RNA-directed DNA methylation, and translational repression [23–25]. Moreover, MITEs also contribute to novel gene formation by providing start sites, poly(A) signals, splicing junctions, and TATA boxes [26, 27].

Pineapple (*A. comosus*) is the most economically important crop possessing crassulacean acid metabolism (CAM) and is a model for studying the evolution of CAM photosynthesis. The pineapple genome has one fewer ancient whole-genome duplication than grass genomes, providing an important reference for tracking evolutionary genomic changes and refining the evolutionary history of grass genomes [28]. In this study, we performed a genome-wide identification and characterization of MITEs in the pineapple genome for a better understanding of genome evolution.

## Results

### Identification and characterization of MITE families in the pineapple genome

**Fig. 1:**
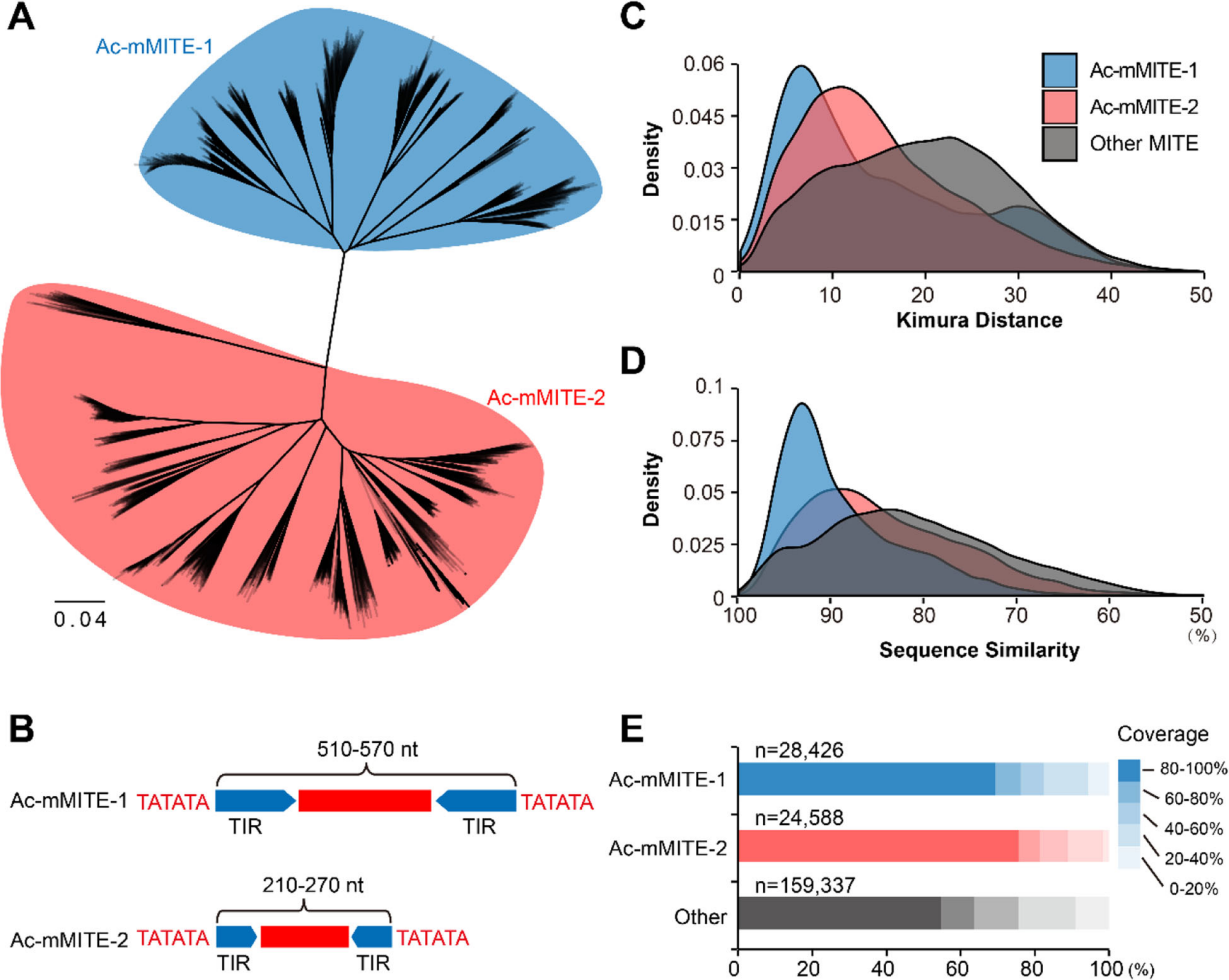
**A** Phylogenetic tree of the full-length Ac-mMITEs. **B** Schematics of the Ac-mMITE-1 and Ac-mMITE-2. **C, D, E** Comparison of Kimura distance (**C**), sequence conservation (**D**), and sequence coverage (**E**) between the two Ac-mMITE families and other MITEs in the pineapple genome

We performed genome-wide identification of MITEs in the pineapple F153 reference genome using MITE-hunter. A total of 4659 representative MITE sequences were identified and they were further grouped into 243 MITE families (Additional file 1: Table S1). The consensus sequences of 243 MITE families (Additional file 2) were imported into RepeatMasker to scan all the associated MITE fragments in the pineapple genome. A total of 212,351 MITE fragments were identified with a total length of 50,210,791 bp, accounting for approximately 13.14% of the pineapple genome. Among these MITE fragments, about 24.41% of them are intact (Table 1; Additional file 1: Table S1). The two largest MITE families, containing 53,014 elements and accounting for 29.39% of the total MITEs and 3.86% of the pineapple genome, were particularly analyzed in this study due to their special flanking sequences (Additional file 1: Table S1). Approximately 74% of them are flanked by TA dinucleotide microsatellites on both sides or one side, and 22 and 16.5% of them are flanked by GA or CT microsatellite, respectively (Additional file 3: Table S2). Therefore, we named these MITEs *A. comosus* microsatellite-associated MITEs (Ac-mMITEs). According to the phylogenetic analysis, Ac-mMITEs were divided into Ac-mMITE-1 and Ac-mMITE-2 (Fig. 1A, B; Additional file 4: Fig. S1), which shared sequence similarity in the terminal inverted repeat (TIR) regions (Additional file 5: Fig. S2; Additional file 6: Fig. S3), suggesting that these two Ac-mMITE families might be derived from a common ancestral or closely related autonomous elements.

**Table 1:** Summary of Ac-mMITEs and other MITEs in the pineapple genome

MITE families	Number of elements (intact elements)	Length (bp)	Total Length (bp)	% in genome	TSD	TIR
**Ac-mMITE-1**	28,426 (9889)	510–570	10,279,806	2.69%	(TA)n	140–180
**Ac-mMITE-2**	24,588 (12,105)	210–270	4,478,432	1.17%	(TA)n	40–70
**Other MITEs**	159,337 (29,851)	70–1300	35,452,553	9.28%	2-10 bp	8–400

In order to gain insight into the evolutionary dynamics of MITEs in the pineapple genome, we calculated Kimura distances (*K*-values) [29], which measure the degree of divergence between TE fragment and consensus. Low *K*-values suggest a relatively recent transposition event and activity. Our result showed that both Ac-mMITE-1 and Ac-mMITE-2 have a lower *K*-value than other MITEs, indicating that Ac-mMITEs have been created by recent transposition events (Fig. 1C). We further compared the sequence conservation and structural integrity of Ac-mMITEs with other MITEs in the pineapple genome. The Ac-mMITEs showed a higher level of sequence similarity and structural integrity than other MITEs (Fig. 1D, E). Taken together, our results imply that Ac-mMITEs have been generated by recent transposition bursts.

### Genomic distribution of Ac-mMITEs

**Fig. 2:**
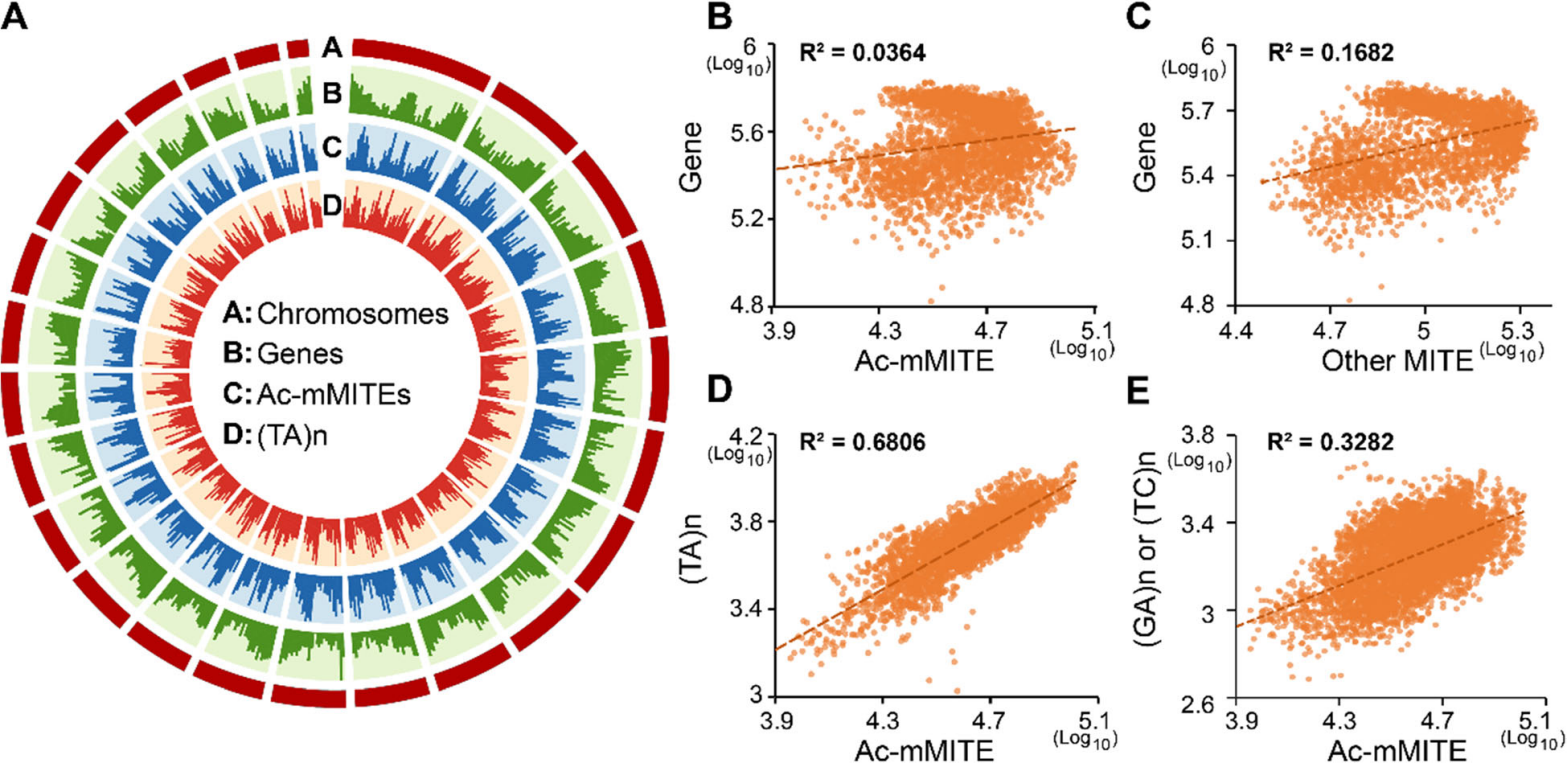
**A** Genomic distribution of genes (**track B**), Ac-mMITEs (**track C**), and (TA) n dinucleotide microsatellite (**track D**) on pineapple chromosomes (**track A**). **B** The Pearson Correlation Coefficients (R^2^) of genome distribution between the genes and Ac-mMITEs. **C** The Pearson Correlation Coefficients (R^2^) of genome distribution between genes and other MITEs. **D** The Pearson Correlation Coefficients (R^2^) of genome distribution between (TA) n microsatellites and Ac-mMITEs. **E** The Pearson Correlation Coefficients (R^2^) of genome distribution between (GA)n/(TC) n microsatellites and Ac-mMITEs

More than 80% of Ac-mMITEs are flanked by dinucleotide microsatellites (Additional file 3: Table S2). We therefore investigated whether dinucleotide microsatellites are preferential target sites of Ac-mMITEs. We observed a strong correlation between the genomic distribution of Ac-mMITEs and (TA) n (R^2^ = 0.6806, Fig. 2A, D), which suggests that (TA) n microsatellites were preferential target sites of Ac-mMITEs. We also observed a positive correlation between the genomic distribution of Ac-mMITEs and (GA) n and (TC) n microsatellites (Fig. 2E), but the R^2^ values are much lower than the one with (TA) n microsatellites. In addition, only 0.20 and 0.10% of Ac-mMITEs are flanked by (GA) n and (TC) n microsatellite on both sides, respectively (Additional file 3: Table S2). Most of the Ac-mMITEs associated with (GA) n and (TC) n microsatellites have (TA) n microsatellite on one side (Additional file 3: Table S2; Additional file 7: Fig. S4). Furthermore, the first and last two bases of the Ac-mMITE consensus sequences are mostly ‘GA’ and ‘TC’ (Additional file 5: Fig. S2). In consistent with this, most (GA) n microsatellites are located at the 5′ end of Ac-mMITEs while most (TC) n microsatellites are located at the 3′ end of Ac-mMITEs (Additional file 3: Table S2; Additional file 7: Fig. S4). All together suggest that (GA) n and (TC) n microsatellites might not be the preferential targets of Ac-mMITEs and the (GA) n and (TC) n microsatellites flanking Ac-mMITEs were likely generated by “DNA replication slippage” after the insertions of Ac-mMITEs.

It has been reported that MITEs preferentially inserted into genic regions and significantly contributed to allelic diversity [30, 31]. We also tested whether there was a correlation of genomic distribution between Ac-mMITEs and genes. The Pearson Correlation Coefficient value calculated between Ac-mMITEs and genes is 0.0364 and much lower than the one calculated between other MITEs and genes (R^2^ = 0.1682) (Fig. 2A, B, C). In addition, the proportion of Ac-mMITEs that are located in intergenic regions is much higher than that of other MITEs, while the proportions of Ac-mMITEs that are located near or within genes are lower than that of other MITEs (Additional file 8: Table S3). Our results suggest that Ac-mMITEs prefer to target gene-sparse regions.

### Ac-mMITEs are related to the *Mutator* superfamily

Among the full-length Ac-mMITEs without dinucleotide microsatellites on both sides or one side, we discovered 1435 of them possess 9/10 bp TSDs. Given the feature of TSDs and TIRs of these Ac-mMITEs, we assumed that the Ac-mMITEs might be derived from *Mutator*-like transposable elements. We searched into pineapple genome to identify the corresponding autonomous elements that provide the transposases required to facilitate transposition of Ac-mMITEs and no such elements were found, suggesting that the related autonomous elements might have largely mutated or degenerated.

**Fig. 3:**
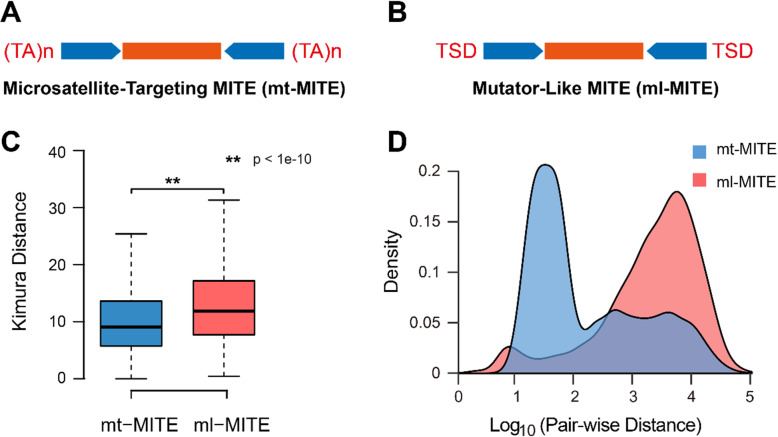
**A** Schematics of the microsatellite-targeting MITEs (mt-MITEs) that are flanked by dinucleotide microsatellites. **B** Schematics of the *Mutator*-like MITEs (ml-MITEs) that contain 9/10 bp TSD. **C** Boxplot displays the Kimura distance of mt-MITEs and ml-MITEs. **D** The adjacent distances of neighboring elements are compared between mt-MITEs (highlighted with blue color) and ml-MITEs (highlighted with red color)

To better understand the evolutionary history of Ac-mMITEs, we filtered Ac-mMITEs based on the features of flanking sequences and performed comparative analysis on the two distinct groups of Ac-mMITEs, microsatellite-targeting MITEs (mt-MITEs) that are flanked by dinucleotide microsatellites on both sides and *Mutator*-like MITEs (ml-MITEs) that contain 9/10 bp TSDs (Fig. 3A, B). The two Ac-mMITE families, Ac-mMITE-1 and Ac-mMITE-2, contained similar proportions of mt-MITEs and ml-MITEs. The copy number of mt-MITEs (15,361) is significantly larger than the ml-MITEs (1435), implying the higher activity of mt-MITEs over the ml-MITEs. Furthermore, the mt-MITEs showed a significantly lower *K*-value than the ml-MITEs (Fig. 3C), which suggests that the mt-MITEs were generated by a more recent amplification burst compared to the ml-MITEs.

### mt-MITEs are frequently clustered via adjacent insertions

We found a large number of intact Ac-mMITEs that are physically close to each other and linked via dinucleotide microsatellites in pineapple genome, indicating that Ac-mMITEs tend to form clusters by adjacent insertions. A cutoff of pair-wise distance of adjacent intact Ac-mMITEs within 100-bp was used to identify the Ac-mMITE clusters in pineapple genome (Additional file 9: Fig. S5). A total of 10,137 full-length Ac-mMITEs, accounting for 46.1% of the total full-length Ac-mMITEs, were screened out, which formed 4024 clusters. Interestingly, the Ac-mMITEs making up these clusters are non-nested and highly variable, indicating that these clusters were formed via multiple independent insertion events, not by tandem duplication. In addition, no identical Ac-mMITE clusters were found in the pineapple genome, supporting that the entire Ac-mMITE cluster may not be capable of transposition. Furthermore, we observed that majority of the adjacent mt-MITEs are located within 100-bp while the ml-MITEs are sparsely distributed by a single unit in the pineapple genome, which is consistent with the discovery that the Ac-mMITE clusters are mostly composed of mt-MITEs (9573/10,137, Fig. 3D).

### Mt-MITEs are highly polymorphic between cultivated pineapples and their wild relatives

**Fig. 4:**
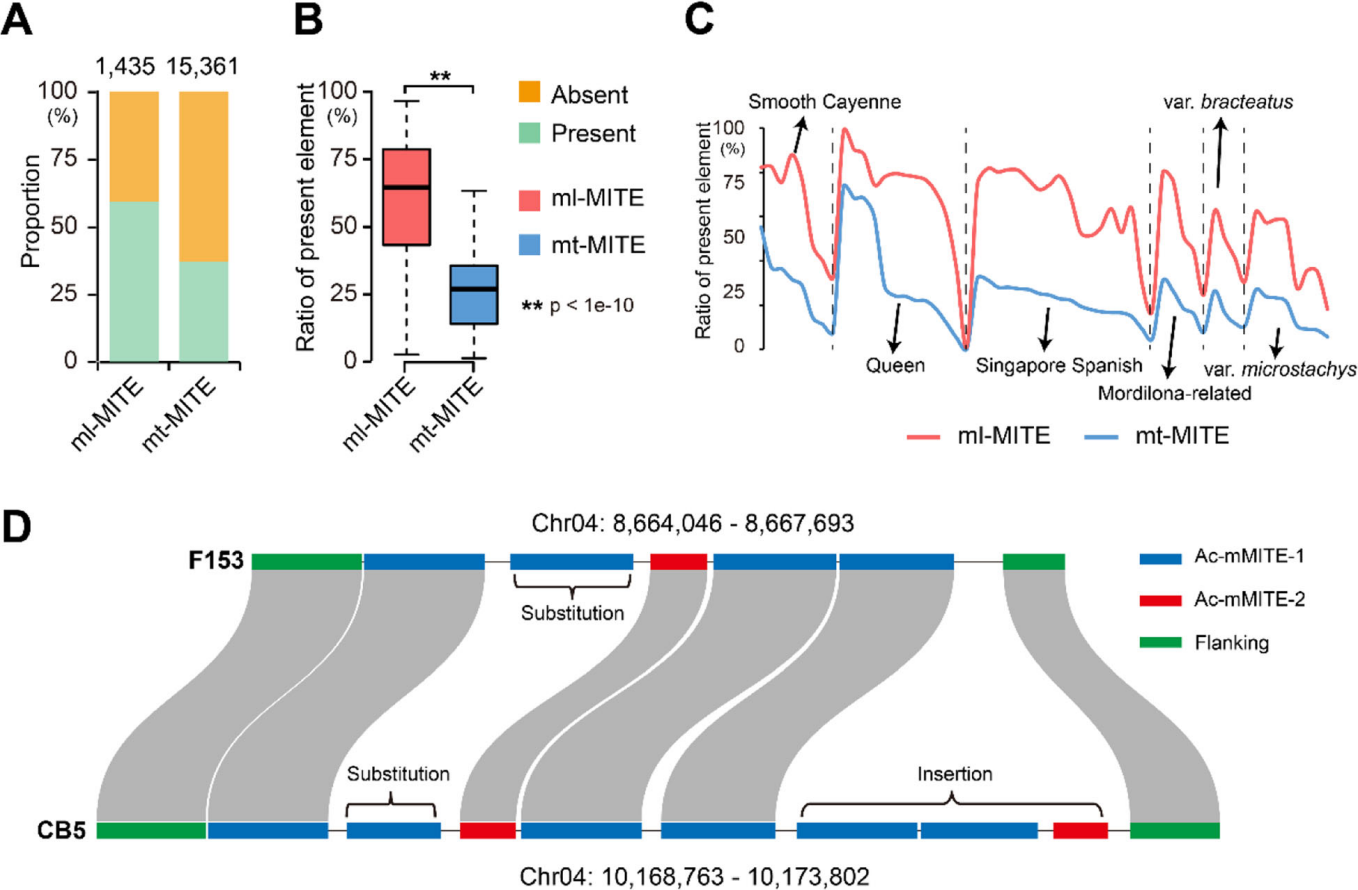
Genome-wide presence and absence variation (PAV) analysis between the cultivated pineapple *A. comosus* var. F153 and its wild relative *A. comosus* var. *bracteatus* CB5, and among 86 *Ananas* accessions. **A** Proportion of present (green) and absent (orange) ml-MITEs and mt-MITEs between F153 and CB5 genomes. **B** Proportion of present ml-MITEs (red) and mt-MITEs (blue) among the 86 *Ananas* accessions. **C** The 86 *Ananas* accessions were divided into six groups based on population structure analysis. Proportions of the present ml-MITEs (red) and mt-MITEs (blue) are displayed. **D** An example shows variations of Ac-mMITE clusters between F153 and CB5 genomes. The coordinates of the Ac-mMITE cluster in F153 and CB5 genomes are labeled above and below of the schematics, respectively

To explore the transposition activity of Ac-mMITEs in the pineapple genome, we performed comparative analysis of Ac-mMITEs between the cultivated pineapple *A. comosus* var. F153 and its wild relative *A. comosus* var. *bracteatus* CB5. Ac-mMITEs account for 3.3% of the CB5 genome, which is at a similar level as in the F153 genome. The sequences of intact Ac-mMITEs in the F153 genome were used as reference to be compared with that in the CB5 genome by performing genome-wide presence and absence variation (PAV) analysis. In total, we discovered 9089 intact Ac-mMITEs, including 5736 mt-MITEs and 851 ml-MITEs, that are present in the CB5 genome. Noticeably, we observed a lower proportion of mt-MITEs than ml-MITEs conserved between the two genomes (37.3% versus 59.3%, Fig. 4A), supporting that the mt-MITEs had experienced more frequent transposition compared to the ml-MITEs after the divergence of the two pineapple varieties from a common ancestor. We further performed PAV analysis using the sequences of the Ac-mMITE clusters in F153 genome as reference and the result revealed that 1123 and 605 clusters, accounting for 28 and 15% of the total clusters, were present and absent in the CB5 genome, respectively. Though the remaining clusters (2296/4024) can be found at the corresponding locations of the CB5 genome, these clusters have exhibited many variations between the two genomes (Fig. 4D). The high variability of these clusters between the two pineapple varieties could be ascribed to random transpositions of mt-MITEs before or after formation of clusters, further demonstrating the recent high activity of mt-MITEs in the pineapple genome.

To further confirm the activity of mt-MITEs, we compared the degrees of insertion polymorphisms between mt-MITEs and ml-MITEs in 86 *Ananas* accessions. Consistent with our assumption, mt-MITEs showed a significantly lower proportion of present orthologous insertions than ml-MITEs (Fig. 4B, C). Based on the structural analysis of the *Ananas* population [32], we divided the *Ananas* accessions into six groups, including four representative groups in the var. *comosus* (‘Queen’, ‘Smooth Cayenne’, ‘Singapore Spanish’, and ‘Mordilona-related’), one group of var. *bracteatus*, and one group of var. *microstachys*. The PAV patterns of Ac-mMITEs among the six groups match their origin and taxonomical relationships. The four groups within the var. *comosus* share a higher level of Ac-mMITEs than var. *bracteatus* and var. *microstachys*. Smooth Cayenne and Queen dispersed from the Guianas, while Singapore Spanish dispersed from the eastern coast of Brazil (south of Bahia) [33]. Smooth Cayenne and Queen groups share a relatively higher level of Ac-mMITEs than the other groups.

### Differential epigenetic regulation of mt-MITEs and ml-MITEs in the pineapple genome

**Fig. 5:**
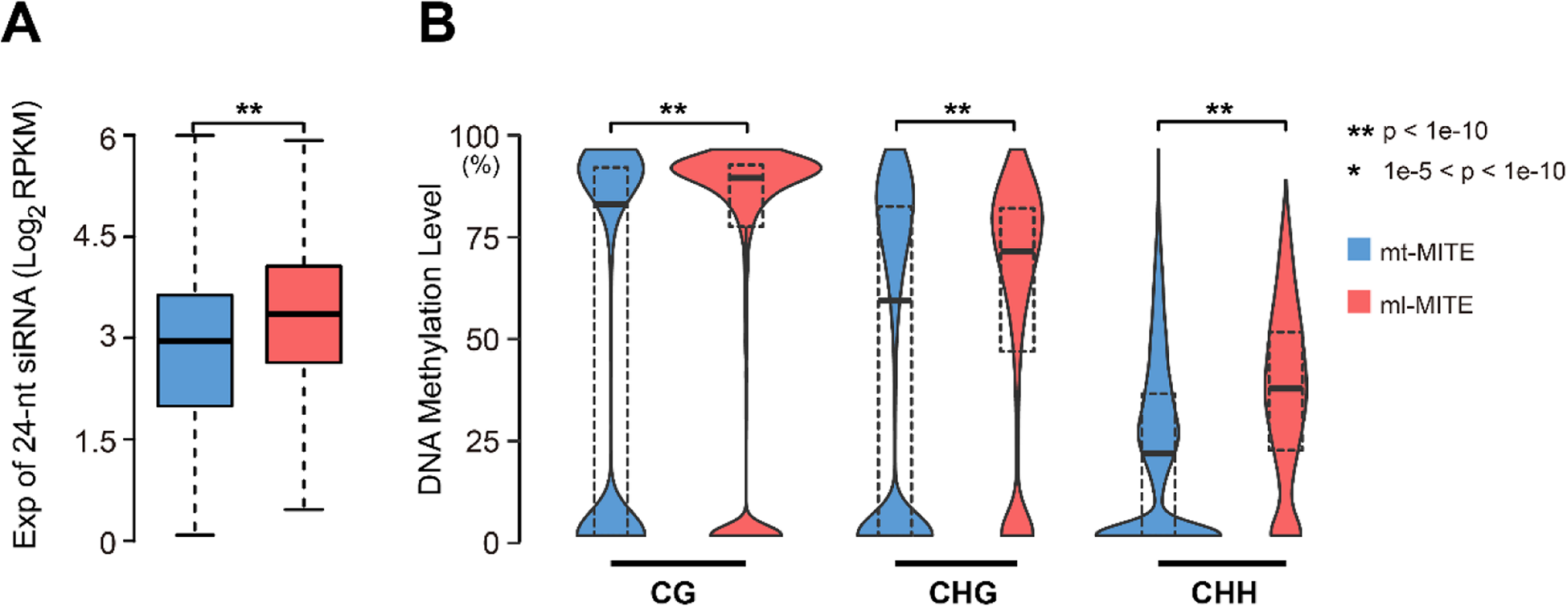
**(A)** Expression levels of 24-nt siRNAs derived from mt-MITEs (blue) and ml-MITEs (red). **(B)** DNA methylation levels of mt-MITEs (blue) and ml-MITEs (red)

Due to the potential deleterious effects of TE insertions, host genomes usually silence TEs epigenetically through small-RNA-mediated DNA methylation to maintain genome integrity [34–36]. We employed the microRNA-seq data [37] (data are available at NCBI BioProject PRJNA311758) and the bisulfite sequencing data [38] (data are available at NCBI BioProject PRJNA493186) to investigate host response and epigenetic regulation of Ac-mMITEs in the pineapple genome. The 24-nt siRNAs derived from mt-MITEs showed a significantly lower level than those from ml-MITEs (Student’s T-test, *p*-value <1e-10, Fig. 5A). In line with this, methylation levels of mt-MITEs were also significantly lower than ml-MITEs (Student’s T-test, p-value <1e-10, Fig. 5B). These results demonstrated the mt-MITEs were not regulated as strictly as the ml-MITEs, which possibly account for the successful amplification of mt-MITE in the pineapple genome.

**Fig. 6:**
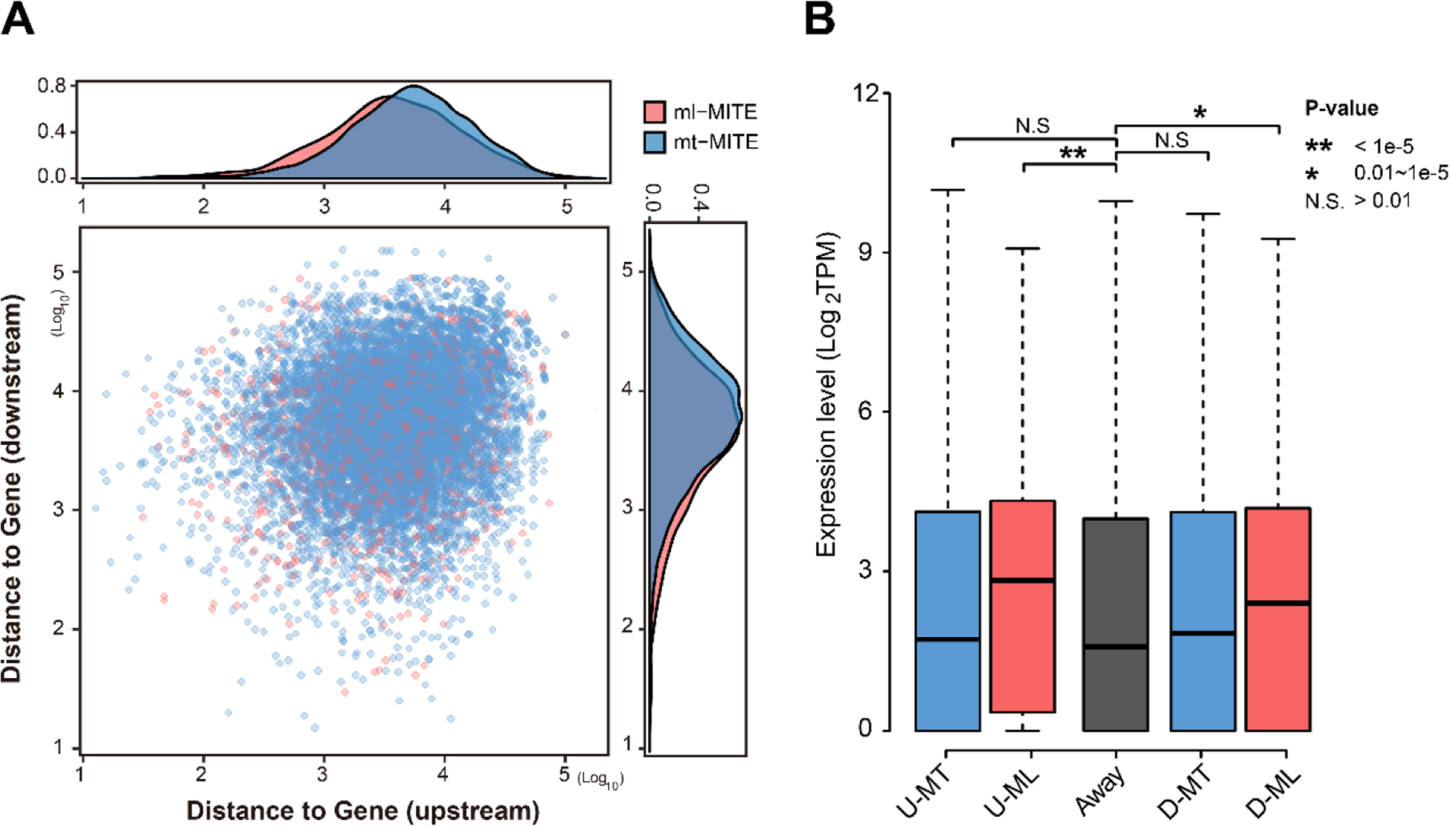
**A)** Dot plot of distance between mt-MITEs (blue) and ml-MITEs (red) and their closest genes on a log_10_ scale. **B)** Comparison of expression levels of five groups of genes. Significance tests are shown on the top

Host genomes counteract TE activity by silencing them epigenetically, but methylation can spread beyond the TE sequence. It has been reported that MITEs have potential impact on gene expression [24, 30, 39]. In rice, genes with embedded or nearby MITEs showed lower levels of expression than the ones without MITE-gene interactions [40]. We discovered a longer distance between mt-MITEs and genes than that between ml-MITEs and genes (Fig. 6A), which is consistent with the lower proportion of mt-MITE assigned in 2-kb flanking regions of genes than that of the ml-MITEs (Table 2). These results suggested that the two kinds of Ac-mMITEs may have different effects on their proximal genes. To validate this assumption, we utilized the pineapple green leaf transcriptomic data (data are available at NCBI BioProject PRJNA493186) and compared expression levels of genes related to mt-MITEs and ml-MITEs separately.

**Table 2:** The associations of mt-MITEs and ml-MITEs with genes

	Total	Intergenic	Upstream	Intron	Downstream
**mt-MITE**	15,361	9120 (59.4%)	2197 (14.3%)	2760 (18.0%)	1922 (12.5%)
**ml-MITE**	1435	672 (46.8%)	346 (24.1%)	265 (18.5%)	261 (18.2%)

In total, we identified 1688 and 1457 genes containing mt-MITE insertion in upstream (named as ‘U-MT’ group) and downstream (named as ‘D-MT’ group) regions, respectively, and 368 and 286 genes possessing ml-MITE insertion in upstream (named as ‘U-ML’ group) and downstream (named as ‘D-ML’ group) regions, respectively. A total of 17,135 genes that do not have Ac-mMITEs nearby (named as ‘AWAY’ group) were used as a reference group. No significant difference in expression levels was observed among U-MT, D-MT and AWAY groups, suggesting that mt-MITEs might have no or negligible impact on nearby gene expression (Student’s T-test, *p*-value > 0.01, Fig. 6B). However, the expression levels of both U-ML and D-ML groups were significantly higher than that of the other groups (Student’s T-test, *p*-value < 0.01, Fig. 6B).

## Discussion

Transposable elements (TEs) constitute a significant fraction of plant genomes and play an important role in genome organization and evolution. Genome-wide identification and characterization of TEs provide essential information for understanding genome structure and evolution. Pineapple is largely vegetatively propagated. Sexual reproduction of pineapple is very rare in nature and is mainly restricted to breeding purpose. TEs might become a major source of genetic innovations in pineapple due to lack of recombination in asexually reproducing organisms [41]. MITEs are short DNA transposons. Although the overall contribution of MITEs to the genome size is small, MITEs usually have high copy numbers [1, 4]. In addition, MITEs play important roles in gene expression and contribute considerable diversity [42].

We performed genome-wide identification and characterization of MITEs in the pineapple genome. The top two most abundant MITE families account for 29.39% of all MITEs and 3.86% of the pineapple genome. Interestingly, approximately 74% of these MITEs are flanked by (TA) n dinucleotide microsatellites, suggesting that they have insertion preference in (TA) n dinucleotide microsatellite regions. Furthermore, these MITEs frequently form non-nested clusters via adjacent insertions and the interval sequences between adjacent elements are almost pure (TA) n microsatellites, reinforcing the hypothesis that (TA) n dinucleotide microsatellite regions are the preferential target sites of Ac-mMITEs.

Mobilization of TEs can be highly mutagenic and cause genomic instability either by direct disruption of normal gene functions or by promoting ectopic homologous recombination, which can lead to harmful genome rearrangements, deletions, and insertions [43, 44]. TEs with seriously deleterious effects on their host genomes will be mostly filtered out by natural selection. Host genomes have also evolved defense mechanisms to suppress TE activities, such as epigenetic silencing [45]. The interaction between TEs and defense mechanisms has led to an evolutionary arms race as well as self-control and targeting mechanism of TEs that mitigate the cost of their propagation on host fitness [46].

TEs are not evenly distributed across the genome and often exhibit various levels of preference of insertion [39, 47]. This may reflect the result of damage-limiting strategy adapted by TEs during co-evolution between TEs and their hosts. The evolutionary success of Ac-mMITEs may lie in their preferential insertion in (TA) n microsatellite regions. A strong bias of TE insertion towards (TA) n microsatellite repeats was also reported in rice [48], *M. truncatula* [49], guayule [50], and mammals [51]. Microsatellite repeats are predominantly non-coding sequences. TE insertion in these regions will have little or no impact on host genome and therefore may protect TEs from genome surveillance systems. In addition, (TA) n microsatellite regions are highly unstable [52], which may facilitate the integration and further transposition of TEs. Disruptions of these vulnerable regions by TE insertion may also increase the stability of these regions and provide potential benefits to host genomes.

The two Ac-mMITE families, Ac-mMITE-1 and Ac-mMITE-2, shared sequence similarity in the distal segments of the terminal inverted repeat (TIR) regions, suggesting that these two Ac-mMITE families were likely derived from a common or closely related autonomous elements. By analyzing the Ac-mMITEs without (TA) n microsatellite flanking sequences, we found that ml-MITEs were likely derived from *Mutator*-like DNA transposon. Ac-mMITEs showed a much higher proportion of intact elements and a lower *K*-value than other MITEs, suggesting that Ac-mMITEs were amplified through recent transposition bursts. mt-MITEs were much more abundant and showed a lower *K*-value than ml-MITEs, suggesting that their preferential insertion in (TA) n microsatellite regions occurred recently and insertion in (TA) n microsatellite regions might have promoted the amplification of mt-MITEs.

Polymorphic insertion analysis revealed highly polymorphic insertion sites of Ac-mMITEs among the 86 *Ananas* accessions. Surprisingly, highly polymorphic insertion sites of Ac-mMITEs were also observed in the close related accessions within the var. *comosus.* Highly divergent insertion of Ac-mMITEs might have resulted from their asexual reproduction and habitat isolation. The 86 *Ananas* accessions share a very low proportion of mt-MITEs. This suggests that mt-MITEs might have been mostly amplified after these accessions separated from a common ancestor and transposition of mt-MITEs might have been ongoing since their divergence.

According to the general senescence patterns of TEs, young TEs are not yet silenced by the host genome and exhibit a low level or no CHH methylation, TEs at intermediate age are effectively silenced and usually show a high level of CHH methylation, and old TEs that are degenerated copies and unable to transpose are no longer silenced by the host genome. Our result showed that the CHH methylation level of mt-MITEs was significantly lower than that of ml-MITEs, providing a different line of evidence to support that mt-MITEs were mostly amplified recently.

TEs play important roles in the evolution of new genes and transcriptome diversity. TE insertions have potential impact on host gene expression through *cis*- or trans- regulatory activities [24, 30, 39, 53, 54]. Studies have implicated MITEs as negative transcription regulators of nearby genes [40]. However, MITEs may also upregulate gene expression by introducing regulatory motifs [39, 53, 54]. ml-MITEs showed a higher level of methylation than mt-MITEs. Surprisingly, genes nearby ml-MITEs showed a higher level of expression than the ones nearby mt-MITEs. Gene expression is controlled at multiple levels. Further studies are needed to address this issue. In general, TE insertions that significantly alter host gene expression patterns will be selected against. Therefore, TEs that cause minimal changes in host gene expression may help them escape the host genome control. Genes with and without mt-MITEs nearby showed similar levels of expression, which may also reflect the result of damage-limiting strategy adapted by mt-MITEs during co-evolution with their host.

## Conclusions

Ac-mMITEs are the most abundant MITEs in the pineapple genome and they were likely derived from *Mutator*-like DNA transposon. Preferential insertion in (TA) n microsatellite regions of Ac-mMITEs occurred recently and is likely the result of damage-limiting strategy adapted by Ac-mMITEs during co-evolution with their host. Insertion in (TA) n microsatellite regions might also have promoted the amplification of mt-MITEs. In addition, mt-MITEs showed no or negligible impact on nearby gene expression, which may help them escape genome control and lead to their amplification.

## Methods

### Identification and classification of MITEs in the pineapple genome

We used the MITE-Hunter program [16] to identify the MITEs in the genome assembly of the pineapple variety F153 [28] with default parameters. The putative MITEs were clustered into different families using VSEARCH 2.6.1 [55] with a parameter of 60% sequence similarity. The two largest MITE families, Ac-mMITE-1 and Ac-mMITE-2, represented by 45 consensus sequences generated by MITE-hunter, were used for further analysis. The flanking sequences of the Ac-mMITEs were manually trimmed using BioEdit [56]. The 45 consensus sequences representing the main subgroups of the Ac-mMITEs were used to scan the F153 genome assemblies using RepeatMasker 4.0.6 with a modified parameter of ‘-nolow -norna -no_is -s -engine crossmatch’. Ac-mMITE fragments with a maximum missing of 10 bp from both terminals compared to the consensus sequences were considered full-length elements (Additional file 10: Fig. S65). The consensus sequences of Ac-mMITE-1 and Ac-mMITE-2 were used to predict the secondary structure of Ac-MITE using RNAstructure 6.0.1 [57, 58].

### Estimation of divergence times

In order to estimate divergence times of Ac-mMITEs, we calculated pairwise Kimura distances [29] between Ac-mMITE copies and their corresponding consensus sequences using RepeatLandscape implemented in RepeatMasker. The transition and transversion rates were calculated on alignments generated by RepeatMasker and transformed to Kimura distance using the following equation: *K* = − 1/2 ln (1 - 2*p* - *q*) - 1/4 ln (1 - 2*q*), where *q* is the proportion of transversion sites and *p* is the proportion of transition sites. We also estimated sequence conservation by calculating similarities between Ac-mMITE sequences and their corresponding consensus sequences using EMBOOSS Needle 6.6.0.0. Structural integrity of Ac-MITEs was also assessed by calculating percent coverage of Ac-mMITEs aligned to their corresponding consensus sequences.

### Construction of phylogenetic tree

To reduce the complexity of the dataset, we selected the top 20% of the full-length Ac-mMITEs with the highest sequence similarity to each of the 45 consensus sequences for constructing bootstrapped neighbor-joining trees using MEGA7 [59]. FigTree 1.4.4 (http://tree.bio.ed.ac.uk/software/figtree/) was used for annotation and final graphic visualization of the phylogenetic tree.

### Mining and characterization of dinucleotide microsatellites (TA) n, (CT) n, and (GA) n in the pineapple genome

We used the Tandem Repeat Finder 4.09 [60] to identify the dinucleotide microsatellites in the pineapple genome by modifying the parameters to ‘2 7 7 80 10 30 2’. Sliding window analysis (500-kb window size, 100-kb steps) was used to analyze the distributions of MITEs, genes, and dinucleotide microsatellites (TA) n, (GA) n, and (TC) n across the pineapple chromosomes, and the results were visualized with Circos 0.69–6 [61].

### Bisulfite sequencing (BS-seq) data analysis

Raw BS-seq reads of pineapple green leaf tip were downloaded from GEO under the accession number of GSE120401 [38]. BS-seq reads were mapped to the F153 reference genome using Bismark 0.20.0 [62] with default settings. The predicted methylation sites with less than 4 or more than 1000 supported reads were removed. The methylation level at each CpG site was obtained by estimating C/(C + T) ratio.

### miRNA-seq and RNA-seq data analysis

Raw miRNA-seq reads of pineapple green leaf were download from NCBI BioProject PRJNA311758 [37] (only the samples collected at 10:00 am were included in this analysis). We used Cutadapt 1.18 [63] to trim the raw miRNA-seq reads. The trimmed reads with length of 24-nt were then extracted and mapped to the pineapple reference genome using Bowtie 1.2.2 [64] with the modified parameters of ‘-v 0 -p 20 -m 2’. The reads that could be mapped to multiple locations were counted reciprocally, and the counted reads were normalized by Reads Per Kilobase per Million mapped reads (RPKMs). Raw RNA-seq reads of pineapple green leaf tip were downloaded from GEO [37] (accession number: GSE120401). We used Bowtie2 2.3.4.1 [65] and RSEM 1.2.29 [66] to map reads and quantify transcripts with default settings. mRNA abundance was then normalized by ‘Transcripts Per Million’ (TPM).

### Ac-mMITE insertion polymorphism analysis

To analyze the presence/absence variations (PAVs) of Ac-mMITEs between the pineapple F153 and CB5 reference genomes, the full-length Ac-mMITEs with 200 bp flanking sequences were extracted from the F153 genome, which was further used as a seed to search into the CB5 genome using NCBI-blastn with a modified parameter of ‘-xdrop_gap 1000 -culling_limit 1 -evalue 1e-100’. An Ac-mMITE was considered ‘present’ in the CB5 genome if the Ac-mMITE with 200 bp flanking sequences can be found at the corresponding position in the CB5 genome with at least 90% sequence similarity. Otherwise, it was marked as ‘absence’.

We further surveyed polymorphisms of the Ac-mMITEs among *Ananas* population of 86 resequencing accessions. The raw reads of 86 *Ananas* NGS data were downloaded from the NCBI BioProject database under the accession number PRJNA389669 [32]. The clean reads were mapped to the F153 genome using Bowtie2 with default parameters. An Ac-mMITE was marked as ‘present’ when there was at least one pair-end reads covering the entire sequence of the Ac-mMITE and 50 bp flanking regions.

To investigate the polymorphisms of Ac-mMITE clusters between the F153 and CB5 genomes, the entire clusters with 200 bp flanking sequences were extracted from the F153 genome and used to run comparative analysis in the CB5 genome. The clusters were considered as ‘present’ in the CB5 genome when: **i**) the number and order of elements are identical. **ii**) the orientation and classification (Ac-mMITE-1 or Ac-mMITE-2) of elements in the cluster are identical; **iii**) each pair of elements display at least 90% of sequence similarity; **iv**) the flanking sequences of the two homologous clusters must have at least 90% of sequence similarity. Otherwise, they were considered ‘absent’ in the CB5 genome. It was defined as a deletion or insertion event when one/few elements absent or one/few additional elements present in a cluster at the corresponding location in the CB5 genome. It was defined as a substitution event when corresponding elements share no or very low sequence similarity or they belong to different Ac-mMITE families.

## Supplementary Information

**Additional file 1: Table S1.** Summary of MITE families in the pineapple F153 genome.

**Additional file 2:** Consensus sequences of all MITE families in the pineapple genome.

**Additional file 3: Table S2.** Summary of flanking sequences of Ac-mMITEs.

**Additional file 4: Figure S1.** Secondary structure analysis of Ac-mMITEs. The consensus sequences of Ac-mMITE-1 **(A)** and Ac-mMITE-2 **(B)** were used to predict secondary structure. The red arrows mark the end of terminal inverted repeats (TIRs).

**Additional file 5: Figure S2.** Alignment of TIR regions of 45 Ac-mMITE-1 and Ac-mMITE-2 consensus sequences. The TIR sequences of the two Ac-mMITE families share sequence similarity at the first 55 bases of 5′ TIR and last 55 bp of the 3′ TIR regions.

**Additional file 6: Figure S3.** Ac-mMITEs show a much higher level of sequence similarity at 5′ and 3′ TIR regions than the middle region. Sequence similarities were calculated based on the 45 consensus sequences representing the main subgroups of Ac-mMITEs using sliding windows of 30-bp windows and 5-bp steps.

**Additional file 7: Figure S4. (A)** Three possible insertions that resulted in Ac-mMITEs flanked by (TA) n (**i**), (GA) n (**ii**) and (CT) n (**iii**). **(B)** Most of the (GA) n are located at 5′ end (**i**) and the (CT) n are located at 3′ end (**iii**) of the Ac-mMITEs.

**Additional file 8: Table S3.** Association of Ac-mMITEs and other MITEs with genes.

**Additional file 9: Figure S5.** The number of Ac-mMITEs with the adjacent distance increasing every 10 bp is shown.

**Additional file 10: Figure S6.** Variations in the two ends of TIRs of the 21,994 intact Ac-mMITEs.

## Data Availability

All the datasets used in this study are publicly available at the National Center of Biotechnology Information (NCBI, http://www.ncbi.nlm.nih.gov/). The pineapple F153 reference genome was published by Ming et al. [25] and the sequence data is available in NCBI GenBank assembly under project PRJNA305080. Resequencing of the 86 *Ananas* accessions was published by Chen et al. [29] and the sequence data is available in NCBI BioProject database under the accession number PRJNA389669. The bisulfite sequencing of pineapple green leaf was published by Shi et al. [35] and the sequence data is available in NCBI GEO database under the accession number GSE120401. The miRNA-seq of pineapple green leaf was published by Wai et al. [34] and the sequence data is available in NCBI BioProject database under the accession number PRJNA311758.

## Abbreviations

MITEs: Miniature inverted-repeat transposable elements
Ac-mMITEs: *A. comosus* microsatellite-associated MITEs
TEs: Transposable elements
TIRs: Terminal inverted repeats
TSDs: Target site duplications
CAM: Crassulacean acid metabolism
mt-MITEs: Microsatellite-targeting MITEs
ml-MITEs: *Mutator*-like MITEs
PAV: Presence and absence variation
