# Supplementary material for: Recent amplification of microsatellite-associated miniature inverted-repeat transposable elements in the pineapple genome
Source: BMC Plant Biol. 2021 Sep 18;21:424. doi: 10.1186/s12870-021-03194-0 (PMC8449440; doi:10.1186/s12870-021-03194-0)
Supplement: Supplementary file 1 — Additional file 1: Table S1. Summary of MITE families in the pineapple F153 genome. [file 12870_2021_3194_MOESM1_ESM.docx]

**Table S1.** Summary of MITE families in the pineapple F153 genome.

| **Superfamily** | **Family Number** | **Copy Number**  **(# of intact elements)** | **Total Length (bp)** | **Fraction in Genome** | **TIR Length (bp)** | **TSD Length (bp)** |
| --- | --- | --- | --- | --- | --- | --- |
| **CACTA** | 5 | 3,334 (1,819) | 1,225,320 | 0.32% | 12-35 | 3/3 |
| **Mutator** | 104 | 58,261 (12,576) | 12,609,437 | 3.30% | 62-1066 | 8/8,9/9,10/10 |
| **hAT** | 48 | 31,648 (6,365) | 7,579,101 | 1.98% | 11-35 | 8/8 |
| **PIF/Harbinger** | 47 | 29,517 (5,401) | 7,918,017 | 2.07% | 8-55 | 3/3 |
| **Ac-mMITE** | 2 | 53,104 (21,994) | 14,758,238 | 3.86% | 40-180 | (TA)n |
| **Unknown** | 37 | 36,577 (3,690) | 6,120,678 | 1.60% | 32-590 | unclassified |
