# Supplementary material for: Recent amplification of microsatellite-associated miniature inverted-repeat transposable elements in the pineapple genome
Source: BMC Plant Biol. 2021 Sep 18;21:424. doi: 10.1186/s12870-021-03194-0 (PMC8449440; doi:10.1186/s12870-021-03194-0)
Supplement: Supplementary file 3 — Additional file 3: Table S2. Summary of flanking sequences of Ac-mMITEs. [file 12870_2021_3194_MOESM3_ESM.docx]

**Table S2.** Summary of flanking sequences of Ac-mMITEs.

|  | **Ac-mMITE-1** | **Ac-mMITE-2** | **Total** | **Fraction** |
| --- | --- | --- | --- | --- |
| **TA/TA** | 3,774 | 4,887 | 8,661 | 39.379% |
| **GA/TA** | 1,307 | 1,960 | 3,267 | 14.854% |
| **TA/TC** | 1,327 | 831 | 2,158 | 9.812% |
| **GA/TC** | 719 | 387 | 1,106 | 5.029% |
| **TA/GA** | 29 | 24 | 53 | 0.241% |
| **GA/GA** | 29 | 22 | 51 | 0.232% |
| **TC/TA** | 18 | 29 | 47 | 0.214% |
| **TC/TC** | 15 | 3 | 18 | 0.082% |
| **TC/GA** | 0 | 0 | 0 | 0.0% |
| **TA/Non** | 423 | 594 | 1017 | 4.624% |
| **GA/Non** | 160 | 274 | 434 | 1.973% |
| **TC/Non** | 2 | 1 | 3 | 0.014% |
| **Non/TA** | 360 | 664 | 1024 | 4.656% |
| **Non/GA** | 1 | 3 | 4 | 0.018% |
| **Non/TC** | 182 | 116 | 298 | 1.355% |
| **Non/Non** | 1543 | 2310 | 3853 | 17.518% |
| Note: 'Non' means non-microsatellite sequence | | | | |
