# Supplementary material for: Recent amplification of microsatellite-associated miniature inverted-repeat transposable elements in the pineapple genome
Source: BMC Plant Biol. 2021 Sep 18;21:424. doi: 10.1186/s12870-021-03194-0 (PMC8449440; doi:10.1186/s12870-021-03194-0)
Supplement: Supplementary file 4 — Additional file 4: Figure S1. Secondary structure analysis of Ac-mMITEs. The consensus sequences of Ac-mMITE-1 (A) and Ac-mMITE-2 (B) were used to predict secondary structure. The red arrows mark the end of terminal inverted repeats (TIRs). [file 12870_2021_3194_MOESM4_ESM.docx]

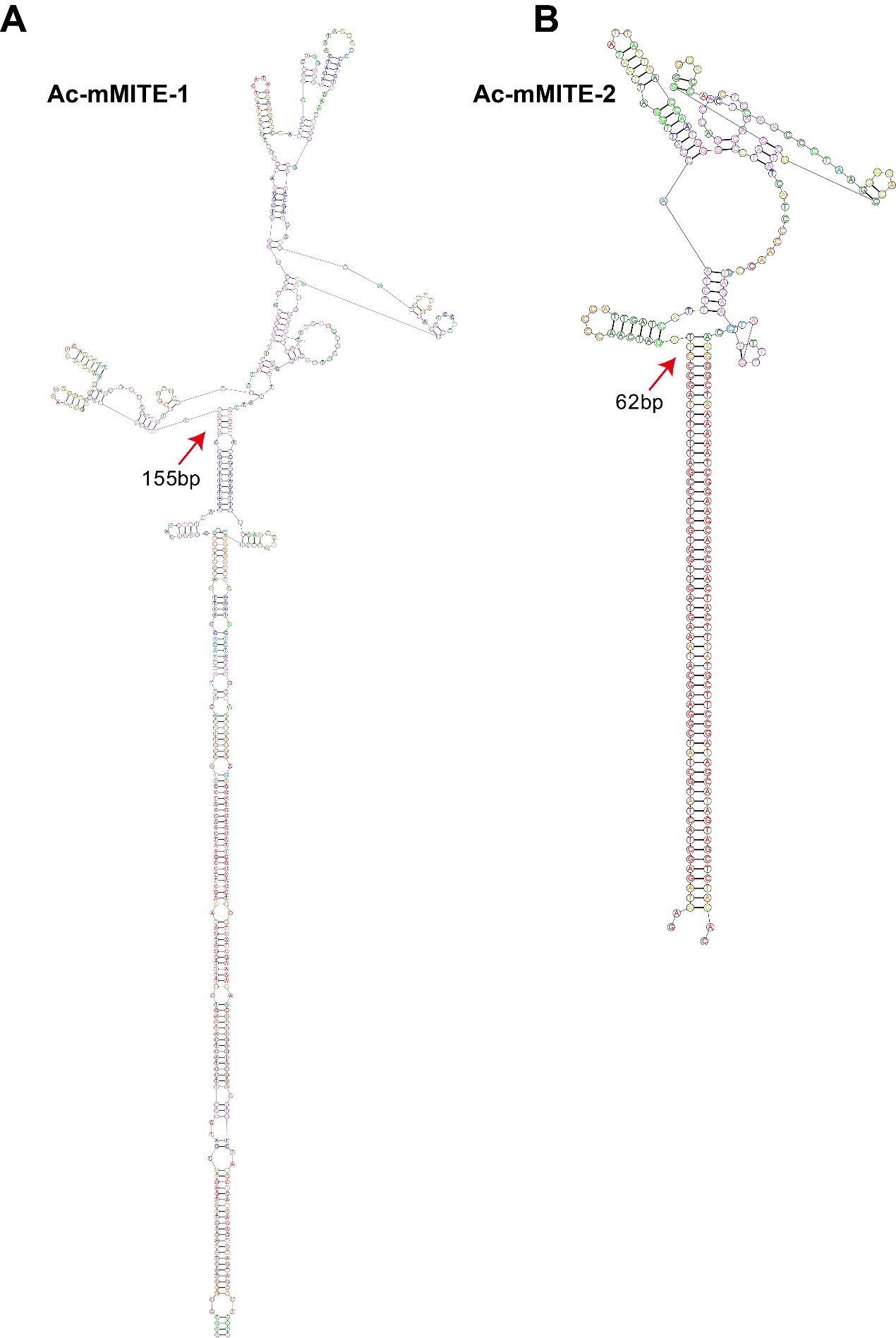


**Figure S1.** Secondary structure analysis of Ac-mMITEs. The consensus sequences of Ac-mMITE-1 (**A**) and Ac-mMITE-2 (**B**) were used to predict secondary structure. The red arrows mark the end of terminal inverted repeats (TIRs).
