# Supplementary material for: Recent amplification of microsatellite-associated miniature inverted-repeat transposable elements in the pineapple genome
Source: BMC Plant Biol. 2021 Sep 18;21:424. doi: 10.1186/s12870-021-03194-0 (PMC8449440; doi:10.1186/s12870-021-03194-0)
Supplement: Supplementary file 5 — Additional file 5: Figure S2. Alignment of TIR regions of 45 Ac-mMITE-1 and Ac-mMITE-2 consensus sequences. The TIR sequences of the two Ac-mMITE families share sequence similarity at the first 55 bases of 5′ TIR and last 55 bp of the 3′ TIR regions. [file 12870_2021_3194_MOESM5_ESM.docx]

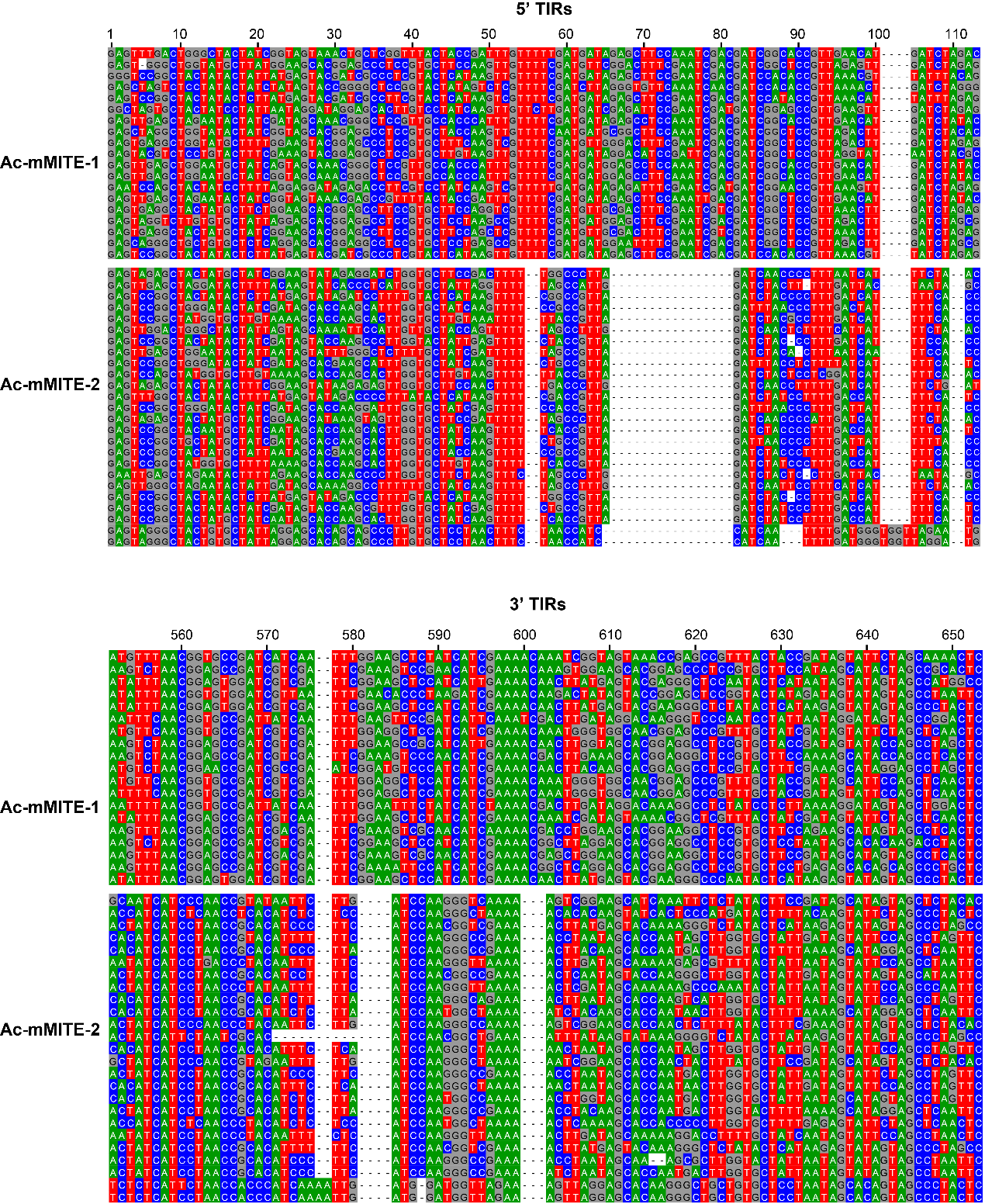


**Figure S2.** Alignment of TIR regions of 45 Ac-mMITE-1 and Ac-mMITE-2 consensus sequences. The TIR sequences of the two Ac-mMITE families share sequence similarity at the first 55 bases of 5’ TIR and last 55 bp of the 3’ TIR regions.
