# Supplementary material for: Recent amplification of microsatellite-associated miniature inverted-repeat transposable elements in the pineapple genome
Source: BMC Plant Biol. 2021 Sep 18;21:424. doi: 10.1186/s12870-021-03194-0 (PMC8449440; doi:10.1186/s12870-021-03194-0)
Supplement: Supplementary file 6 — Additional file 6: Figure S3. Ac-mMITEs show a much higher level of sequence similarity at 5′ and 3′ TIR regions than the middle region. Sequence similarities were calculated based on the 45 consensus sequences representing the main subgroups of Ac-mMITEs using sliding windows of 30-bp windows and 5-bp steps. [file 12870_2021_3194_MOESM6_ESM.docx]

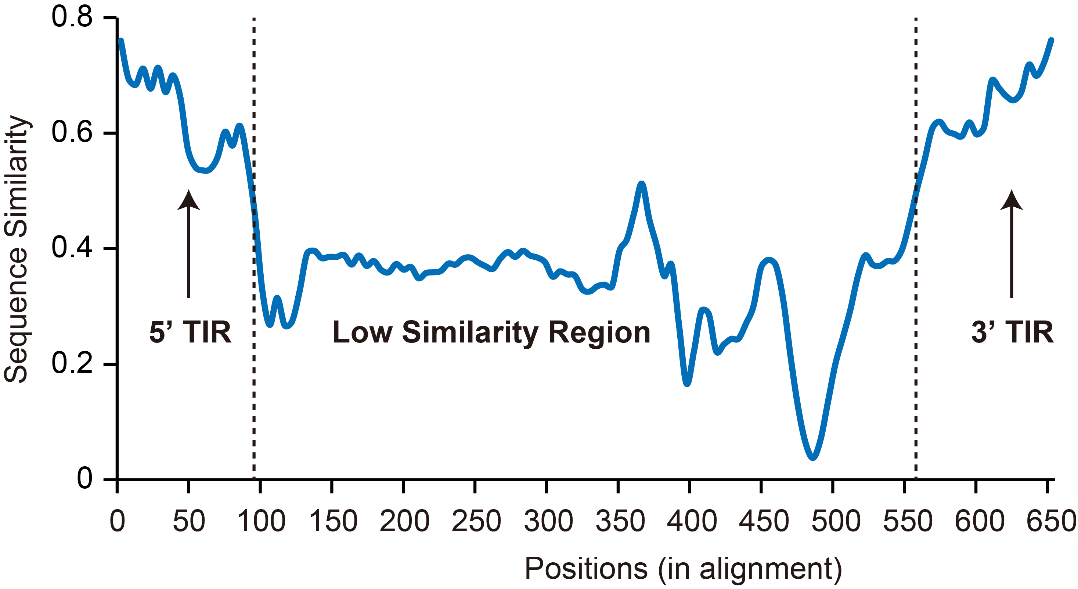


**Figure S3.** Ac-mMITEs show a much higher level of sequence similarity at 5’ and 3’ TIR regions than the middle region. Sequence similarities were calculated based on the 45 consensus sequences representing the main subgroups of Ac-mMITEs using sliding windows of 30-bp windows and 5-bp steps.
