# Supplementary material for: Recent amplification of microsatellite-associated miniature inverted-repeat transposable elements in the pineapple genome
Source: BMC Plant Biol. 2021 Sep 18;21:424. doi: 10.1186/s12870-021-03194-0 (PMC8449440; doi:10.1186/s12870-021-03194-0)
Supplement: Supplementary file 7 — Additional file 7: Figure S4. (A) Three possible insertions that resulted in Ac-mMITEs flanked by (TA) n (i), (GA) n (ii) and (CT) n (iii). (B) Most of the (GA) n are located at 5′ end (i) and the (CT) n are located at 3′ end (iii) of the Ac-mMITEs. [file 12870_2021_3194_MOESM7_ESM.docx]

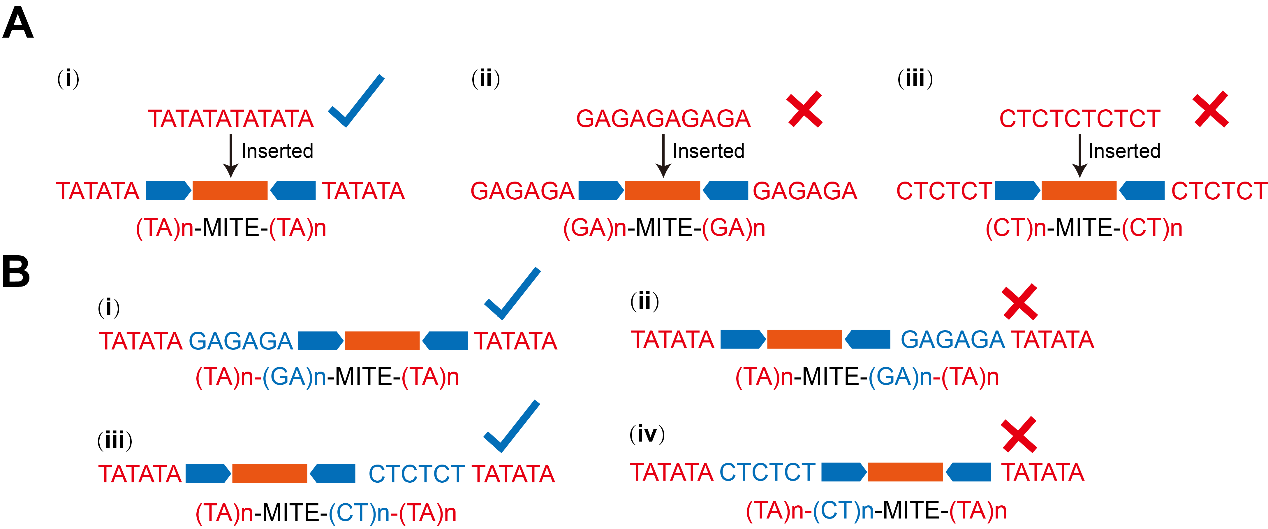


**Figure S4. (A)** Three possible insertions that resulted in Ac-mMITEs flanked by (TA)n (**i**), (GA)n (**ii**) and (CT)n (**iii**). **(B)** Most of the (GA)n are located at 5’ end (**i**) and the (CT)n are located at 3’ end (**iii**) of the Ac-mMITEs.
