# Supplementary material for: Recent amplification of microsatellite-associated miniature inverted-repeat transposable elements in the pineapple genome
Source: BMC Plant Biol. 2021 Sep 18;21:424. doi: 10.1186/s12870-021-03194-0 (PMC8449440; doi:10.1186/s12870-021-03194-0)
Supplement: Supplementary file 8 — Additional file 8: Table S3. Association of Ac-mMITEs and other MITEs with genes. [file 12870_2021_3194_MOESM8_ESM.docx]

**Table S3.** Association of Ac-mMITEs and other MITEs with genes.

|  | **Total Number** | **> 2kb from genes** | **< 2kb from genes*** | **Within genic regions** |
| --- | --- | --- | --- | --- |
| **Ac-mMITEs** | 53,014 | 30,060 (56.7%) | 14126 (26.6%) | 10189 (19.2%) |
| **Other MITEs** | 159,337 | 70,941 (44.5%) | 60462 (37.9%) | 34229 (21.5%) |

*The category “Within genic regions” is included in the category “< 2kb from genes”.
