# Supplementary figures and images for: Recent amplification of microsatellite-associated miniature inverted-repeat transposable elements in the pineapple genome
Source: BMC Plant Biol. 2021 Sep 18;21:424. doi: 10.1186/s12870-021-03194-0 (PMC8449440; doi:10.1186/s12870-021-03194-0)

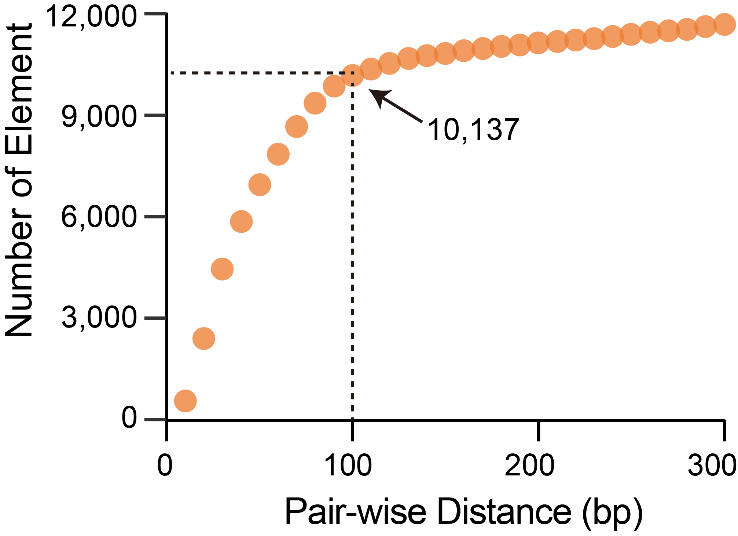


**Figure S5.** The number of Ac-mMITEs with the adjacent distance increasing every 10 bp is shown.

Supplement: Supplementary file 9 — Additional file 9: Figure S5. The number of Ac-mMITEs with the adjacent distance increasing every 10 bp is shown. [file 12870_2021_3194_MOESM9_ESM.docx]

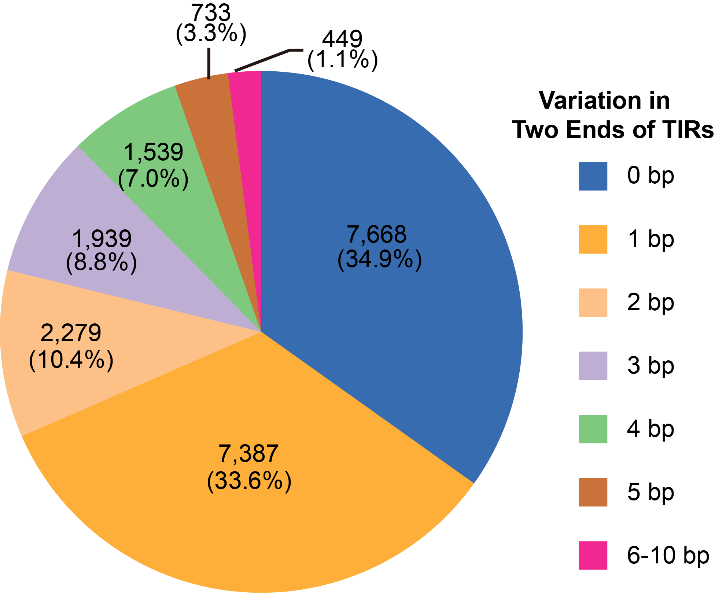


**Figure S6.** Variations in the two ends of TIRs of the 21,994 intact Ac-mMITEs.

Supplement: Supplementary file 10 — Additional file 10: Figure S6. Variations in the two ends of TIRs of the 21,994 intact Ac-mMITEs. [file 12870_2021_3194_MOESM10_ESM.docx]
